# The natural history of secondary muscle-invasive bladder cancer

**DOI:** 10.1186/1471-2490-13-23

**Published:** 2013-05-08

**Authors:** Guy Hidas, Dov Pode, Amos Shapiro, Ran Katz, Liat Appelbaum, Galina Pizov, Kevin C Zorn, Ezekiel H Landau, Mordechai Duvdevani, Ofer N Gofrit

**Affiliations:** 1 Department of Urology, Hadassah University Medical Center, Jerusalem, Israel; 2 Department of Radiology, Hadassah University Medical Center, Jerusalem, Israel; 3 Department of Pathology, Hadassah University Medical Center, Jerusalem, Israel; 4 Department of Surgery, Section of Urology, University of Montreal Hospital Center, Montreal, Canada; 5 Department of Urology, Hadassah University Medical Center, PO Box 12000, Jerusalem, 91120, Israel

**Keywords:** Bladder cancer, Muscle invasive, Progression, Survival, Outcomes, Accuracy, BCG, Intravesical therapy

## Abstract

**Background:**

The management of patients with high-grade non muscle invasive bladder cancer (NMIBC) brings diagnostic and therapeutic challenges. In the current study, we sought to study the natural history of progression to "secondary" muscle-invasive bladder cancer (MIBC)-cancer that developed during follow up of patients presenting with non-muscle invasive bladder cancer (NMIBC).

**Methods:**

Between 1998 and 2008, 760 patients were treated for bladder cancer. Primary MIBC (>=T2) tumors (present upon presentation) were diagnosed in 114 patients. All patients with high-grade NMIBC were treated with intravesical BCG. Mean follow-up was 44 months.

**Results:**

Forty patients (6.1%) developed secondary MIBC after a mean period of 21 months from initial diagnosis of bladder cancer. The 2- and 5-year disease-specific survival rates were better for patients with secondary MIBC (90% and 56% compared to 69% and 42% for patients with primary disease, p=0.03). The Kaplan-Meier curves of the two groups were parallel but displaced by approximately 2 years.

**Conclusion:**

In the current series, MIBC progression occurred among initially presenting patients with NMIBC in 6.1%. In most patients, the initial diagnosis of NMIBC is correct and muscle invasion occurs after a mean period of about 2 years. This supports a non-radical approach in patients with high-grade T1, Ta or Tis. Meticulous follow-up with liberal biopsy of any suspicious lesion may provide early diagnosis of invasive disease.

## Background

Approximately 25% of the urothelial tumors have invaded the detrusor muscle upon presentation (stage ≥T2), the rest are NMIBC confined to the bladder mucosa (Ta) or lamina propria (T1) [1]. High-grade T1 tumors share many morphologic, genetic and clinical characteristics with muscle invasive cancer, including varying degrees of aggressiveness and lethality potential [2-4]. These tumors tend to recur in 40-70% and progress to muscle invasion in 10-20% of cases [5,6].

The management of patients with T1 disease is one of the most challenging issues in urologic oncology. Transurethral resection followed by intravesical immunotherapy with bacillus Calmete-Gúerin is currently advised [7]. Initial response to this therapy is as high as 80%, but over a ten-year period, only 30% of these patients remain free of tumor progression or recurrence and up to 50% require radical cystectomy. Disease-specific mortality rate in these patients is as high as 30% [4,8].

Although aggressive, radical cystectomy for T1 disease provides a 5-year disease-specific survival outcome of 80% in reported series [9-11]. Due to morbidity and disfigurement associated with cystectomy, the timing of radical cystectomy for high-risk T1 patients remains controversial.

Approximately 85% of the patients with MIBC present with muscle-invasion at the first diagnostic procedure (primary cases), while the rest have a history of NMIBC (secondary cases). Although patients with primary and secondary muscle invasive disease are both offered radical cystectomy the prognosis of these patients is unclear with contradictory results in the literature.

Worse prognosis of patients with secondary T2 disease compared to primary T2 was reported [12]. Other authors reported on similar prognosis [13,14] and even better prognosis for secondary T2 [15]. All these studies are limited by a small number of patients and by the inclusion of patients who underwent radical cystectomy only. Patients with T2 who were treated non-operatively were not included in any of these studies.

Outcomes analysis of all patients with primary and secondary T2-bladder cancer may provide data regarding the accuracy of TURBT staging and the natural history of high-grade bladder cancer. To the best of our knowledge, this has never been reported.

## Methods

### Patients and treatment

Information was obtained from the Hadassah Hebrew University Hospital database for 760 patients treated for bladder cancer between 1998 and 2008. This retrospective study was approved by the Institutional Review Board Committee (Institutional Helsinki committee, IRB number 207–31.10.08).

Diagnoses of primary and secondary bladder cancer were made using TURBT histological specimens. Pathologic staging was performed according to the TNM system and grading according to the 1973 WHO classification [16]. All specimens were reviewed by a dedicated uropathologist. Patients with high-grade tumors on initial diagnosis underwent a second look TURBT 4–6 weeks following initial resection. When the diagnosis of T1, Tis or high grade Ta was confirmed an induction course of 6 weekly intravesical instillations of 81 mg Connaught BCG in 50 cc of normal saline was initiated 10–20 days following the TURBT. Six weeks following the last instillation, urinary cytology and cold-cup bladder biopsies were obtained. A second induction course was offered to patients with disease persistence. Responders were given maintenance therapy (2–3 instillations every 3 months for one year and 2–3 instillations every 6 months for additional 2 years). Follow-up protocol included urinary cytology and cystoscopy every 3 months for 2 years and every 6 months for an additional 3 years. Upper tract surveillance (intravenous pyelogram in most instances) was done annually in patients with high-grade disease.

The diagnosis of MIBC initiated metastatic workup which included computer tomography of the chest abdomen and pelvis. Patients with no evidence of metastases and reasonable risk for general anesthesia were offered radical cystectomy. Neoadjuvant chemotherapy was not used in any patient.

### Statistical analysis

Disease-free survival for patients with primary MIBC was calculated from diagnosis. For patients with secondary MIBC, survival was calculated twice, from the initial diagnosis of bladder cancer and from diagnosis of MIBC. Differences in survival between patients with primary and secondary MIBC were calculated using Kaplan-Meier method. Continuous variables were compared using *t*-test and categorical variables using Fischer’s exact test and the chi square test. A p-value <0.05 was considered statistically significant.

## Results

760 patients (mean age 67.5 years, S.D. 12.7 years, 137 females and 623 males) were treated for bladder cancer at our institution including 476 (62.6%) patients with Ta tumors, 55 (11.5%) of whom had high-grade carcinoma. 157 patients presented with T1 disease (all with high-grade cancer) and 114 with primary MIBC disease. Tis was diagnosed in 137 patients including 13 patients with pure Tis and 124 patients with concomitant Tis. Metastatic disease was present in 10 patients upon presentation, and these patients were excluded from the analysis.

A total of 40 patients with NMIBC progressed to MIBC during a mean follow up of 44 months (range=2-150 months) (secondary MIBC). This group included 30 patients with initial T1, 5 patients with initial pure Tis and 5 patients with initial Ta disease. Median time to progression was 12 month (range 3 to 132 month). Patients with secondary MIBC were compared to 104 patients with primary muscle invasive bladder cancer in the first TURBT.

Table 1 compares the characteristics of patients with primary and secondary muscle invasive disease. There was a trend that patients with secondary MIBC were younger than patients with initial diagnosis of invasive disease (69.3 versus 72.7, p=0.08). Radical cystectomy was performed in 64 patients (61.5%) of the primary group and 23 patients (57.5%) in the secondary group. The principle reason for not proceeding with radical cystectomy were patient preference (36 patients) and medical comorbidity (21 patients).

**Table 1:** Demographic details of patients with primary and secondary muscle-invasive bladder cancer

	**Primary**	**Secondary**	**P value**
Number	104	40	
Mean Age years (S.D.)	72.7 (11.0)	69. 3 (11.3)	0.08
Sex (%)			
Male	79 (75.9)	33 (82.5)	0.5
Female	25 (24.1)	7 (17.5)	
Number of Patients who underwent Cystectomy (%)	64 (61.5%)	23 (57.5%)	0.13
Mean Follow-up in months (range)	40.1 (2–150)	52.6 (5–150)	0.08

Pathological outcomes following radical cystectomy are summarized in Table 2. The T-stage distribution of patients with primary and secondary MIBC was significantly different (p=0.014), with higher incidinces of pT0 and pT3 disease in patients with secondary MIBC. N0 was more common among patients with secondary disease (91.3%) compared to primary cases (79.6%) however this trend was not statistically significance (p=0.33).

**Table 2:** Final pathological staging of patients with primary and secondary muscle-invasive bladder cancer who underwent cystectomy

**Pathological stage**	**Primary**	**Secondary**
pT0	5 (7.8%)	4 (17.4%)
pT1	4 (6.2%)	1 (4.3%)
pT2	25 (39%)	4 (17.4%)
pT3	30 (46.9%)	11 (47.8%)
pT4	0	3 (13%)
N0	51 (79.7%)	21 (91.3%)
N+	13 (20.3%)	2 (8.7%)

**Figure 1:**
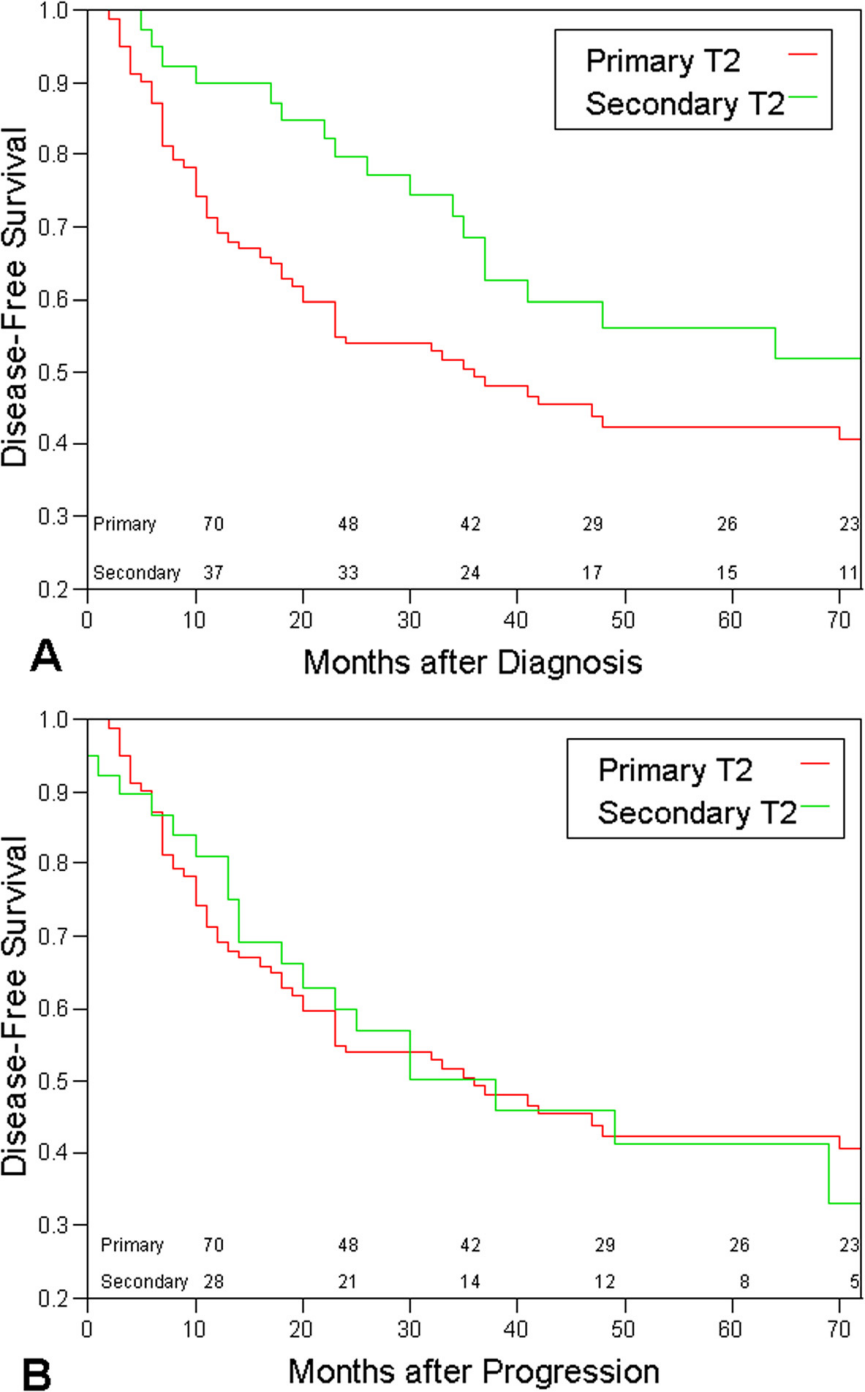
**Kaplan-Meier survival curves comparing the disease specific survival of patients with primary and secondary T2 bladder cancer.** The numbers represent the numbers of patients at risk. **A**- Disease specific survival is calculated from the day of bladder cancer. **B**- Disease specific survival is calculated from the day of progression to T2 bladder cancer.

Figure 1A presents a Kaplan-Meier analysis of disease specific survival comparing patients with primary and secondary MIBC. Survival was calculated from time of initial bladder cancer diagnosis. The 2- and 5- year disease specific survival rate were 69% and 42%, respectively for patients with primary muscle-invasive tumors and 90% and 56%, respectively for patients with secondary muscle-invasive tumors (p=0.03). The curve of patients with secondary T2-disease parallels the curve of the primary T2-deferred by approximately two years.

Figure 1B presents Kaplan-Meier analysis of the same groups of patients, but survival was calculated from time of progression to muscle invasive disease. The two curves cross each other multiple times. The 2- and 5- year specific survival rate of patients who progressed were 81% and 41% respectively (p=0.91).

Analysis of the sub-population that underwent cystectomy also shows that patients with secondary T2 have better prognosis (2 and 5-year disease free survival of 95% and 66% Vs. 85% and 60% in the secondary and primary cases respectively), however, without statistical significance, p=0.45).

## Discussion and conclusions

The natural history of high-grade NMIBC is difficult to predict. Rapid progression to muscle invasion and metastasis in some patients and indolent, non-progressive course in others are well known. Factors known to reflect tumor aggressiveness include high histological grade, presence of carcinoma-in- situ and tumor recurrence following BCG induction [15]. Despite intense research, clinically useful biological markers for predicting prognosis are still absent [7].

The management of high-grade tumors, especially high-grade T1 cancer is still controversial. Complete transurethral resection of the tumor, second look and adjuvant intravesical installation of Bacillus Calmete-Guerin (BCG) are usually recommended and remain the standard of care [7]. This policy is supported by Sylvester et al. who performed meta-analysis of 24 randomized clinical trails including 4863 patients and showed that induction and maintenance BCG treatment significantly reduces the risk of superficial bladder cancer progression by 27% (from 13.8% to 9.8% p=0.001)[17]. Moreover, other authors have demonstrated long-term survival benefit for more aggressive approaches, specifically early radical cystectomy for the high risk T1 urothelial cancer [2,11,18,19].

In the current study, we have observed that after median follow-up of 44 months (3.75 years), disease specific survival of patients with secondary MIBC is not inferior, rather, superior to the disease specific survival of patients with primary MIBC (90% vs 69% 2-year disease specific survival, p=0.03).

Interestingly, the disease specific survival curves of patients with primary and secondary MIBC shows that they are parallel, however the curve for the secondary MIBC cases is displaced by about 2 years. When disease specific survival of patients with secondary MIBC is calculated from the date of progression to MIBC, the survival of patients with primary and secondary MIBC is similar (Figure 1B).

Several previous comparisons of patients with primary and secondary MIBC showed contradictory results [12-15]. Schrier et al., observed poorer outcomes in patients with secondary MIBC (3 years disease specific survival of 67% and 37% for patients with primary and secondary MIBC respectively, p=0.0015) [12]. Both May et al. [13] and Ferreira et al. [14] showed similar prognosis of patients with primary and secondary MIBC. The five years overall survivals were 46% and 52% for primary MIBC and 50% and 58% for patients with secondary T2 in the studies of May et al. and Ferreira et al. respectively. Vaidya et al. on the other hand, showed survival benefit for patients with secondary MIBC, with a 2 years disease free survival of 49% in the primary group and 79% in the secondary invasive group [15].

It is difficult to explain the differences between the studies. Different population composition can explain some of discrepancy. In the study by Schrier et al. [12] for instance, patients with secondary tumors were 5 years older than patients with primary tumors, while in the current study and in the study by Vaidya et al., patients with secondary disease were younger than patients with primary MIBC disease. Additionally, the rate of positive lymph nodes in the work of Schrier et al. was very high in both groups (30% in the primary and 28% in the secondary cases) while only 20% of the primary cases and 8.6% of the secondary cases had positive lymph nodes in the current study. This suggests that delayed treatment was given to patients in both groups reported in the study of Schrier et al. Additionally, there is the difference in study design between the current and previous studies. While all previous studies were based on analysis of cystectomy registries, the current study is based on analysis of cancer registry and also includes patients who were not operated.

The significant clinical question behind the figures is whether a patient diagnosed with high-grade NMIBC and later progresses to MIBC misses the opportunity to be cured by early radical cystectomy due to metastatic spread.

The results presented here are encouraging and support the policy of conservative treatment with strict follow up strategy for several reasons: (i) They show that the initial diagnosis of T1 bladder cancer is correct in most cases (when confirmed by "2^nd^ look" biopsies). Muscle invasion (if it occurs) is a later event. (ii) Even if progression to T2 occurs, many patients can still be salvaged by delayed cystectomy (or by other treatments), and their prognosis is not compromised compared to patients with primary T2 (iii) The lead time between stage T1 and stage T2 is about 2 years. Meticulous follow up during this period and a liberal use of bladder biopsies of any suspicious lesion can potentially diagnose muscle invasion early.

The current study is limited by being retrospective. Answering the same question in a prospective study however, requires randomizing patients with high-grade NMIBC to early and late cystectomy groups. This design seems highly unacceptable by most patients.

In conclusion, according to the results, in most cases the initial diagnosis of T1 disease is accurate and progression occurs later on. Patients with high-grade NMIBC are properly managed by intra-vesical instillation of BCG and by meticulous follow-up. The time interval between the diagnosis of NMIBC and muscle invasion is about two years.

## Competing interests

The authors declare that they have no competing interests.

## Authors’ contributions

GH, AS, and ONG designed the research; GH, and ONG conducted the research; GP provided the pathological database; LA provided the radiological database; GH, DP, KCZ, EHL and MD analyzed the data; GH and ONG wrote the paper; ONG had the primary responsibility for the final content; All authors read and approved the final version.

## Pre-publication history

The pre-publication history for this paper can be accessed here:

http://www.biomedcentral.com/1471-2490/13/23/prepub
